# Supplementary material for: Comparative multiomics analysis of cell physiological state after culture in a basket bioreactor
Source: Sci Rep. 2022 Nov 23;12:20161. doi: 10.1038/s41598-022-24687-4 (PMC9686226; doi:10.1038/s41598-022-24687-4)
Supplement: Supplementary file 1 — Supplementary Information 1. [file 41598_2022_24687_MOESM1_ESM.zip › raw data/Transcriptomic raw data/6.Enrichment/2.KEGG/TE1vsTC1/ALL/TE1vsTC1.all_KEGGbar.pdf]

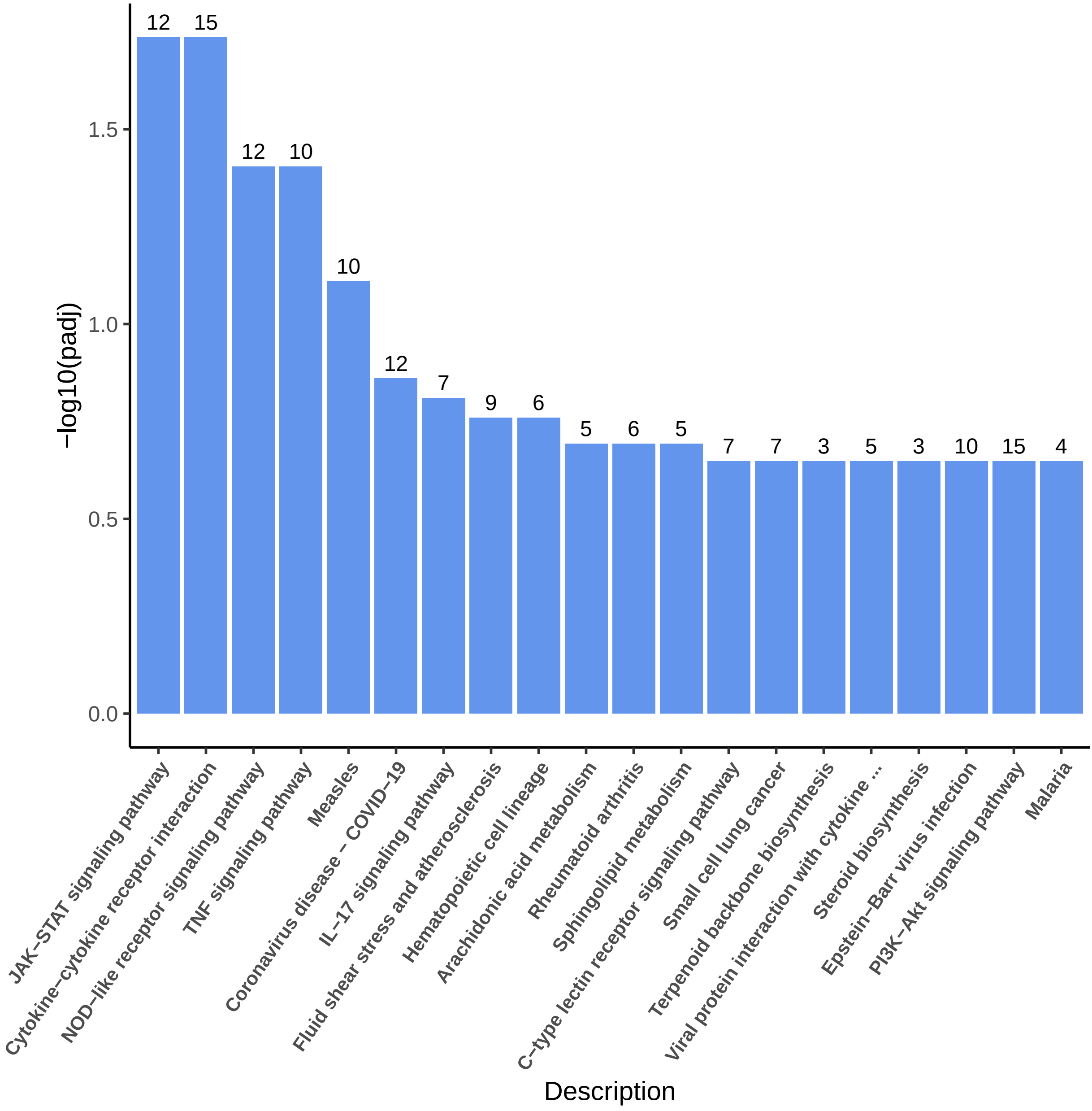

-log10(padj)
1.5
1.0
0.5
0.0
12
15
12
10
10
12
7
9
6
5
6
5
7
7
3
5
3
10
15
4
JAK-STAT signaling pathway
Cytokine-cytokine receptor interaction
NOD-like receptor signaling pathway
TNF signaling pathway
Measles
Coronavirus disease - COVID-19
IL-17 signaling pathway
Fluid shear stress and atherosclerosis
Hematopoietic cell lineage
Arachidonic acid metabolism
Rheumatoid arthritis
Sphingolipid metabolism
C-type lectin receptor signaling pathway
Small cell lung cancer
Terpenoid backbone biosynthesis
Viral protein interaction with cytokine ...
Steroid biosynthesis
Epstein-Barr virus infection
PI3K-Akt signaling pathway
Malaria
Description
